# New Medical Journals

**Published:** 1859-03

**Authors:** 


NEW MEDICAL JOURNALS.—We place with pleasure the following new journals, which have appeared since the first of the present year, on our exchange list:—The *New York Medical Press*, edited by Drs. Kiernan and O'Meagher; the *Semi-monthly Medical News*, edited by Drs. Bemiss and Benson, of Louisville; and the *Louisville Medical Gazette,* edited by Dr. L. F. Frazee.

